# Maresin-1 reduces the pro-inflammatory response of bronchial epithelial cells to organic dust

**DOI:** 10.1186/1465-9921-14-51

**Published:** 2013-05-10

**Authors:** Tara M Nordgren, Art J Heires, Todd A Wyatt, Jill A Poole, Tricia D LeVan, D Roselyn Cerutis, Debra J Romberger

**Affiliations:** 1 VA Nebraska-Western Iowa Healthcare System, Research Service, Omaha, NE, USA; 2 Pulmonary, Critical Care, Sleep & Allergy, University of Nebraska Medical Center, Omaha, NE, USA; 3 Department of Environmental, Agricultural, and Occupational Health, University of Nebraska Medical Center, Omaha, NE, USA; 4 Department of Epidemiology, University of Nebraska Medical Center, Omaha, NE, USA; 5 Department of Oral Biology & Pharmacology, Creighton University Medical Center, Omaha, NE, USA

**Keywords:** Maresin-1, Pro-resolving mediators, Organic dust exposures, Airway inflammation, Bronchial epithelial cells

## Abstract

**Background:**

Exposure to organic dust causes detrimental airway inflammation. Current preventative and therapeutic measures do not adequately treat resulting disease, necessitating novel therapeutic interventions. Recently identified mediators derived from polyunsaturated fatty acids exhibit anti-inflammatory and pro-resolving actions. We tested the potential of one of these mediators, maresin-1 (MaR1), in reducing organic dust-associated airway inflammation.

**Methods:**

As bronchial epithelial cells (BECs) are pivotal in initiating organic dust-induced inflammation, we investigated the *in vitro* effects of MaR1 on a human BEC cell line (BEAS-2B). Cells were pretreated for 1 hour with 0–200 nM MaR1, followed by 1–24 hour treatment with 5% hog confinement facility-derived organic dust extract (HDE). Alternatively, a mouse lung slice model was utilized in supportive cytokine studies. Supernatants were harvested and cytokine levels determined via enzyme-linked immunosorbent assays. Epithelial cell protein kinase C (PKC) isoforms α and ϵ, and PKA activities were assessed via radioactivity assays, and NFκB and MAPK-related signaling mechanisms were investigated using luciferase vector reporters.

**Results:**

MaR1 dose-dependently reduced IL-6 and IL-8 production following HDE treatment of BECs. MaR1 also reduced HDE-stimulated cytokine release including TNF-α in a mouse lung slice model when given before or following HDE treatment. Previous studies have established that HDE sequentially activates epithelial PKCα and PKCϵ at 1 and 6 hours, respectively that regulated TNF-α, IL-6, and IL-8 release. MaR1 pretreatment abrogated these HDE-induced PKC activities. Furthermore, HDE treatment over a 24-hour period revealed temporal increases in NFκB, AP-1, SP-1, and SRE DNA binding activities, using luciferase reporter assays. MaR1 pretreatment did not alter the activation of NFκB, AP-1, or SP-1, but did reduce the activation of DNA binding at SRE.

**Conclusions:**

These observations indicate a role for MaR1 in attenuating the pro-inflammatory responses of BECs to organic dust extract, through a mechanism that does not appear to rely on reduced NFκB, AP-1, or SP-1-related signaling, but may be mediated partly through SRE-related signaling. These data offer insights for a novel mechanistic action of MaR1 in bronchial epithelial cells, and support future *in vivo* studies to test MaR1’s utility in reducing the deleterious inflammatory effects of environmental dust exposures.

## Background

Agricultural-related organic dust exposures are known to trigger airway inflammation. Individuals working within environments such as concentrated animal feeding operations (CAFOs) experience chronic respiratory diseases associated with this work [1,2]. Airway inflammation resulting from organic dust exposure is characterized by heightened pro-inflammatory cytokine release, neutrophil infiltration, and tissue remodeling processes. Long-term effects of chronic exposure include increased risk for lung function loss and obstructive pulmonary diseases [3]. Preventative measures such as the use of respirator masks to limit dust exposure are available, although these measures are not widely adopted or consistently utilized amongst the exposed populations [4]. Currently available therapeutics do not adequately treat or alleviate disease [5]. Therefore, improved preventative and therapeutic options are needed to assist this population of affected individuals.

To develop better-tailored therapies to prevent or treat organic dust-related airway inflammation, the basic biology underlying the disease must be more fully investigated. Previously published research using organic dust extracts derived from hog CAFOs (HDE) has shown a single exposure to organic dust causes bronchial epithelial cells (BECs) to release early response cytokines (tumor necrosis factor-α [TNFα], interleukin-6 [IL-6], and interleukin-8 [IL-8] *in vitro* or keratinocyte-derived chemokine [KC] and macrophage inflammatory protein-2 [MIP-2]) *in vivo*, leading to the recruitment of pro-inflammatory neutrophils and macrophages [6]. This pro-inflammatory HDE-stimulated cytokine release in BECs requires the activation of PKC [7,8]. Inhibiting the activation of pathways associated with these pro-inflammatory processes in BECs may alleviate the subsequent detrimental lung inflammation.

Specialized pro-resolving lipid mediators (SPM) such as resolvins, lipoxins, protectins, and maresins are derived from the metabolism of polyunsaturated fatty acids (PUFAs) [9-11]. These mediators have been shown to exhibit a variety of cell type-specific anti-inflammatory and pro-resolving effects, as reviewed by G. Bannenberg in 2010 [12]. These effects include reducing neutrophil infiltration, polarizing macrophages to an M2 phenotype while increasing phagocytic capacities, modulating pro-inflammatory cytokine release by epithelial cells and promoting neutrophil clearance across mucosal surfaces. While reducing inflammation, SPM have also been shown to increase lung immunity and resistance to infection [13,14]. These attributes make lipid mediators favorable candidates for treating pulmonary diseases characterized by neutrophil-emphasized inflammatory processes, such as those associated with organic dust exposures.

The potential utility of PUFAs or PUFA-derived mediators has not yet been studied in the context of organic dust exposures, although published literature reporting their use in other similar models of inflammation has demonstrated a potential therapeutic application. For example, resolvin E1 has shown promise in reducing pro-inflammatory cytokine release, improving host immunity in the context of acute lung injury, and reducing cell infiltration and airway hyper-responsiveness in a murine model of asthma [14-16]. Resolvin D1 has been reported to promote resolution of airway inflammation induced by cigarette smoke as well as acute lung injury caused by LPS in mice [16,17]. Although not yet studied in the lung environment, MaR1 has shown utility in reducing neutrophil infiltration while increasing macrophage phagocytic capacities in a murine model of peritonitis [18]. Previously published data from our group suggest the recruitment and subsequent actions of macrophages in organic dust exposures are highly important in determining the outcomes of the pro-inflammatory insult, and pro-inflammatory cytokine production by BECs exposed to injurious stimuli such as organic dusts is key to the recruitment of these macrophages as well as neutrophils into the lung [19-23]. However, MaR1’s effects on BECs, along with other cells in the lung are currently unknown.

In consideration of the important roles that BECs play in potentiating the pro-inflammatory effects of HDE, including the release of cytokines, in part through protein kinase C (PKC) isoform activation, and recruitment and activation of other leukocyte responders [7,24-26], the purpose of our study was to determine whether MaR1 would reduce the pro-inflammatory effects in BECs induced by HDE. Endpoint measurements included PKCα and PKCϵ activities, transcription factor binding activities and cytokine release. Results of the experiments reported here demonstrate that MaR1 can reduce dust-induced PKCα and PKCϵ activation and pro-inflammatory cytokine release in the BECs. While NFκB, AP-1, and SP-1-related signaling are important to the pro-inflammatory response of BECs to HDE, MaR1 pretreatment did not reduce the DNA-binding activities of these transcription factors. Although, the HDE-induced DNA binding activities at the serum response element (SRE) is reduced upon MaR1 pretreatment, suggesting this pathway is modified by MaR1. Taken together, these data reveal previously uncharacterized anti-inflammatory effects of MaR1 on BECs and *ex vivo* mouse lung slice cultures exposed to HDE and support future *in vivo* studies testing the utility of MaR1 for potential treatment of organic dust-mediated lung inflammation.

## Methods

### Materials

7(S)-Maresin-1 (7S,14R-dihydroxy-4Z,8E,10Z,12Z,16Z,19Z-docosahexaenoic acid) and 7(R)-Maresin-1 (7R,14S-dihydroxy-4Z,8E,10E,12Z,16Z,19Z-docosahexaenoic acid) were obtained from Cayman Chemical (Ann Arbor, MI, USA). The human bronchial epithelial cell line BEAS-2B was purchased from American Type Culture Collection (Manassas, VA, USA).

### Animal care and housing

Male C57Bl/6 mice were purchased from Jackson Laboratories (Bar Harbor, ME, USA) and housed in cages (group housing) under pathogen-free conditions. Mice received a standard mouse chow diet, and care was supervised by the University of Nebraska Medical Center Animal Care Facilities. Experimental use of animals was regulated and approved by the University of Nebraska Medical Center Institutional Animal Care and Use committee.

### Preparation of mouse lung slices

Mouse lung slices were prepared using previously described methodology [27,28]. Briefly, C57BL/6 male mice were euthanized with 50 mg/mL pentobarbital, and lungs filled with low melting point agarose. Lungs were sliced using a vibrating microtome (EMS-4000; Electron Microscope Sciences, Hatfield, PA) and cultured for 4 days in RPMI medium (with 2 medium changes) prior to use in experiments.

### Tissue culture

BEAS-2B cells were grown as submerged cultures in serum-free LHC-9 (Invitrogen; Grand Island, NY):RPMI (Sigma; St. Louis, MO, USA) media (1:1) containing 100 U/ml Penicillin + 100 μg/ml Streptomycin (Invitrogen; Grand Island, NY, USA). Cells were incubated at 37°C/5% CO_2_ and passaged via trypsinization. Experiments were performed using cells of approximately 85% confluency.

### Preparation of organic dust extract

Organic dust extract was prepared as previously described [7]. Briefly, settled dust from hog confinement facilities was placed in Hanks’ balanced salt solution (Biofluids; Rockville, MD, USA) (1 gram dust per 10 ml solution). This solution was incubated for 1 hour, followed by two vortexing and centrifugation steps. The resulting supernatant was sterile-filtered (0.2 μM filter) (Nalgene; Rochester, NY, USA) and aliquoted at −20°C.

### TNF-α, IL-6, and IL-8/CXCL1 cytokine levels

BEAS-2B cells were pretreated for 1 hour with 0–200 nM 7(S)-MaR1 or 7(R)-MaR1, followed by 5% HDE for 24 hours. Cell supernatants were collected and IL-6 and IL-8 enzyme-linked immunosorbent assays (ELISAs) were performed as previously described [7]. Alternatively, mouse lung slices were pre-treated for 1 hour with 0–200 nM MaR1, followed by 5% HDE treatment, or given 5% HDE treatment followed by 0–200 nM 7(S)-MaR1 1 hr after the HDE treatment was given. At 24 hours following HDE treatment, lung slice supernatants were collected and assayed for murine TNF-α, IL-6, and the murine IL-8 cognate CXCL1 using ELISAs.

### Transcription factor binding activities

Cells were reverse transfected onto 96-well plates using Cignal Vector Reporters for NFκB, AP-1, SP-1, and SRE (SABiosciences; Valencia, CA, USA), using manufacturer’s directions with Lipofectamine 2000 (Invitrogen; Grand Island, NY, USA). Transfected cells were treated with 5% HDE (with or without 0–200 nM 7(S)-MaR1 pretreatment) for 1–24 hours, then harvested using Promega Dual-Glo Luciferase Reagent (Promega; Madison, WI, USA); luciferase activity was measured on a Victor 3 V plate reader (Perkin Elmer; Waltham, MA, USA). The luciferase vectors work such that a firefly luciferase reading that is inducible (i.e. AP-1 reporter) is obtained, as well as a constitutive renilla luciferase reading, for normalization. Normalized data is thus expressed as relative luciferase units, which are compared across treatment groups and expressed as fold-change values over controls.

### PKCα, PKCϵ, and PKA activities

BEAS-2B cells were pre-treated for 1 hour with 0, 100, or 200 nM 7(S)-MaR1, followed by 1 hour or 6 hours treatment with 5% HDE. Cells were lysed and measured for PKCα and PKCϵ kinase activities, as previously described [29-31].

### Statistical analyses

Student’s *t* tests and ANOVA (Tukey’s method for post-hoc multiple comparisons) tests were used to compare control versus treated groups, as appropriate. P values ≤ 0.05 were considered significant. Data are expressed as mean +/− standard error of the mean (SEM). Graphing/statistical analyses were performed using the Graphpad Prism software program.

## Results

### MaR1 reduces the release of HDE-induced pro-inflammatory cytokines (IL-6 and IL-8) by airway epithelial cells (BEAS-2B cell line)

**Figure 1:**
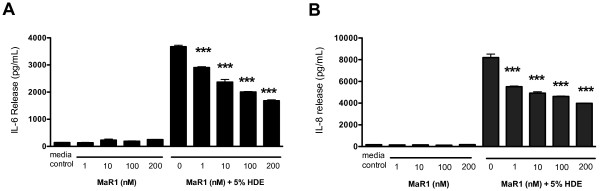
**Effects of MaR1 pretreatment on HDE-induced IL-6 and IL-8 release 24 hours following HDE treatment in bronchial epithelial cells.** BEAS-2B cells were pretreated with 0, 1, 10, 100, or 200 nM MaR1 for 1 hour, then treated with 5% HDE. At 24 hours post HDE treatment, cell supernatant was collected and assayed for **A**) IL-6 and **B**) IL-8 levels, using ELISAs. ***p < 0.001 compared to 5% HDE. Data is representative of 4 separate experimental replicates (biological replicates), with two technical replicates per experiment.

Previous studies have shown treatment of BECs with 5% HDE for 24 hours leads to significant increases in IL-6 and IL-8 release [7,8]. To test the effects of MaR1 pretreatment on cytokine release from BECs in response to HDE, BEAS-2B cells were pretreated with 0, 1, 10, 100, or 200 nM MaR1 (in all studies, the 7[S] form of MaR1 was used, unless otherwise stated). After 1 hr, 5% HDE was added to the cultures and allowed to incubate for 24 hours. In cells receiving MaR1 pretreatment, IL-6 and IL-8 cytokine levels were dose-dependently inhibited compared to cells receiving HDE alone, as measured at 24 hours post-HDE exposure (Figure 1). In the absence of HDE, MaR1 alone had no effect on IL-6 or IL-8 levels at any concentration tested. These studies were repeated using the 7(R) isoform of MaR1, achieving similar results (data not shown).

### MaR1, given prior to or during HDE treatment, reduces the release of pro-inflammatory cytokines (TNF-α, IL-6, and CXCL1) in a mouse lung slice model of HDE exposure

**Figure 2:**
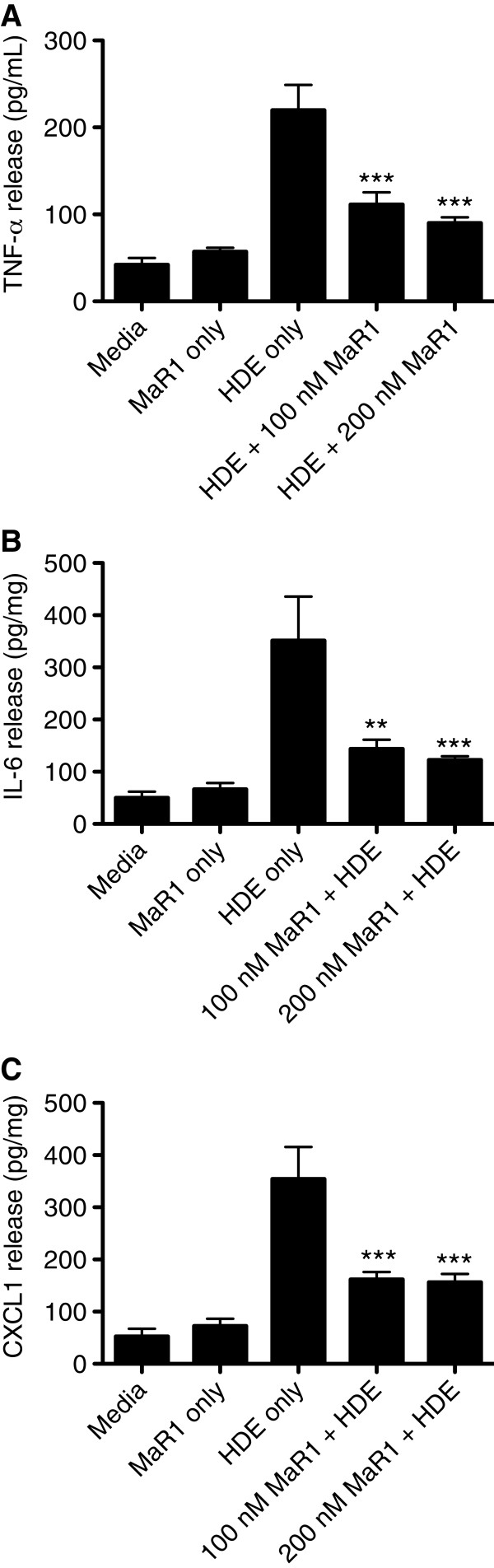
**Effects of MaR1 pretreatment on HDE-induced TNF-α, IL-6, and CXCL1 release 24 hours following HDE treatment in mouse lung slices.** Mouse lungs slices were pre-treated for 1 hour with 0, 100, or 200 nM MaR1, followed by 5% HDE treatment. At 24 hours following HDE treatment, lung slice supernatants were collected and assayed for murine TNF-α (**A**)*,* IL-6 (**B**), and the murine IL-8 cognate CXCL1 (**C**). **p < 0.01 vs. 5% HDE; ***p < 0.001 vs. 5% HDE. Data are representative of 4 separate experimental replicates.

**Figure 3:**
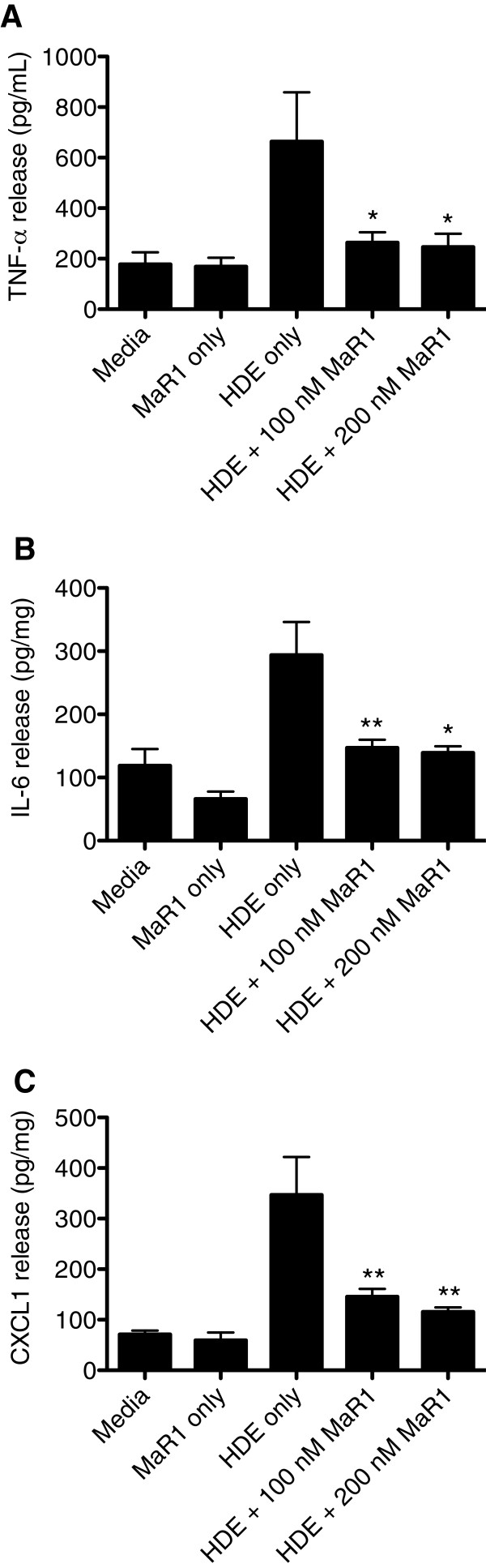
**Effects of MaR1 treatment given during HDE exposure on HDE-induced TNF-α, IL-6, and CXCL1 release 24 hours following HDE treatment in mouse lung slices.** Mouse lungs slices were given 5% HDE treatment. At 1 hour following the addition of HDE, 0, 100, or 200 nM MaR1 was added to the slices. At 24 hours following HDE treatment, lung slice supernatants were collected and assayed for murine TNF-α (**A**)*,* IL-6 (**B**), and the murine IL-8 cognate CXCL1 (**C**). *p < 0.05 vs. 5% HDE; **p < 0.01 vs. 5% HDE. Data are representative of 3 separate experimental replicates.

To ascertain the reproducibility of MaR1’s effects in a translationally relevant airway model system, mouse lung slices were used to test the capacity of MaR1 to limit HDE-induced cytokine release. Using the mouse lung slice model, precision cut mouse lungs slices prepared from C57Bl/6 mice were pretreated for 1 hour with 0, 100, or 200 nM MaR1 followed by 5% HDE. Alternatively, lung slices were first treated with 5% HDE, then given 0, 100, or 200 nM MaR1concomitantly with HDE treatment (at 1 hour following treatment with HDE). At 24 hours following HDE treatment, TNF-α, IL-6, and CXCL1 (murine IL-8 homolog) cytokine release were significantly inhibited in a dose-dependent manner in MaR1 pre-treatment (Figure 2) and MaR1 post-treatment (Figure 3) studies. Taken together, these results show the ability of MaR1 to attenuate the release of the pro-inflammatory HDE-induced TNF-α, IL-6, and CXCL1 cytokines as both a pretreatment as well as after initial HDE exposure (MaR1 given after introduction of HDE). Additionally, these results provide validation for the effects of MaR1 on HDE-stimulated cytokine release by BEAS-2B in culture.

### Effects of HDE with and without MaR1 pretreatment on PKCα, PKCϵ, and PKA kinase activities

**Figure 4:**
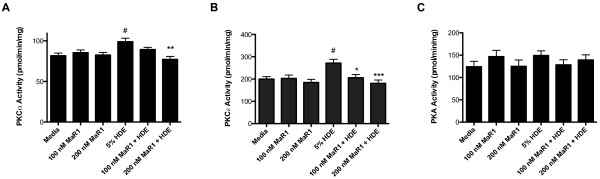
**Effects of MaR1 pretreatment on HDE-induced PKCα, PKCϵ, and PKA activities following treatment.** BEAS-2B cells were pretreated with 0, 100, or 200 nM MaR1 for 1 hr, then treated with 5% HDE. At 1 hr or 6 hr post HDE treatment, cells were flash frozen, then assayed for PKCα (1 hr; **A**), PKCϵ (6 hr; **B**), and PKA (1 hr; **C**) activities, as previously described. #p < 0.05 vs. media; *p < 0.05 vs. 5% HDE; **p < 0.01 vs. 5% HDE; ***p < 0.001 vs. 5% HDE. PKA and PKC isoform-specific activities experiments were performed 3 separate times.

Data from our group has shown HDE augments the release of TNF-α, IL-6, and IL-8 from cultured airway epithelial cells and *in vivo* in a manner that is dependent, at least in part, on PKC activation [8]. Specifically, sequential activation of PKCα and PKCϵ isoforms occurs following HDE exposure, with PKCα activity peaking at 1 hour and PKCϵ activity peaking at 6 hours following HDE exposure [7,8]. In dominant-negative PKCα BECs, TNF-α is not produced, and PKCϵ activation, IL-6, and IL-8 release are abrogated. However, in PKCϵ dominant-negative BECs, TNF-α and IL-6 release are not affected, but IL-8 release is diminished [8]. These studies highlight the importance of PKC activation in the release of TNF-α, IL-6, and IL-8 in HDE-stimulated BECs. Therefore, to determine the effects of MaR1 on kinases that regulate the HDE-induced pro-inflammatory responses, BEAS-2B cells were pretreated with 0, 100, or 200 nM MaR1 for 1 hour, followed by 1 or 6 hour treatment with 5% HDE. Cell lysates were then assayed for PKCα and PKCϵ kinase activity levels. As expected, in cells treated with 5% HDE, PKCα levels were significantly increased within 1 hour following HDE exposure. Pretreatment with 100 or 200 nM MaR1 eliminated this effect (Figure 4A). Similarly, in BEAS-2B cells treated for 6 hours with 5% HDE, PKCϵ kinase activity was significantly stimulated over untreated controls, while maresin-1 pretreatment eliminated this effect (Figure 4B). MaR1 did not inhibit phorbol ester (PMA)-stimulated PKCα or PKCϵ, suggesting that MaR1 is not functioning as a non-specific kinase inhibitor of these enzymes (data not shown). These results indicate MaR1 can reduce the activation of PKCα and PKCϵ during HDE stimulation of BECs. Because PKC activity is known to potentiate the pro-inflammatory responses in BECs exposed to HDE, MaR1’s mechanism of reducing inflammatory cytokine release is likely mediated in part through these inhibitory actions on PKCα and PKCϵ.

Previous experiments by our laboratory have shown that PKA activation negatively regulates the pro-inflammatory cytokine release in BECs induced by HDE exposure [32]. We therefore sought to determine whether or not MaR1 was inhibiting the release of pro-inflammatory cytokines not only by inhibiting PKCα and PKCϵ activity, but also through the activation of PKA. To determine the effects of MaR1 on PKA, we pretreated BEAS-2B cells with 0, 100, or 200 nM MaR1 for 1 hour, then treated with 5% HDE for 1 hour. Cell lysates were then assayed for PKA activity. We found that MaR1 did not significantly alter PKA activity levels when given as a pretreatment to HDE for 1 hour (Figure 4C). Similarly, no change in PKA activity was observed after 6 hour HDE exposure in MaR1-pretreated cells (data not shown). These data suggest that the inhibition of PKCα and PKCϵ and concomitant reduction in IL-6 and IL-8 release by BECs following HDE stimulation by MaR1 is independent of the actions of PKA.

### Effect of HDE on transcription factor binding activities in BECs with and without MaR1 pretreatment

**Figure 5:**
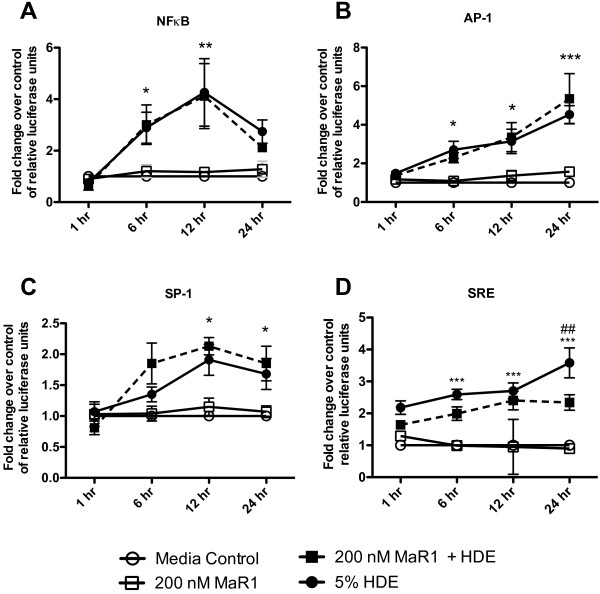
**Effects of HDE on transcription factor binding activity in bronchial epithelial cells.** BEAS-2B cells were treated with 0 or 200 nM MaR1 for 1 hr, then treated with 5% HDE. At 1, 6, 12, and 24 hours following HDE treatment, cells were harvested and assayed for NFκB (**A**), AP-1 (**B**), SP-1 (**C**), and SRE (**D**) transcription factor binding activities using SABiosciences dual luciferase vector systems. *p < 0.05 vs. media; **p < 0.01 vs. media; ***p < 0.001 vs. media. ## p < 0.01 vs. HDE. Data are shown as a fold change of relative luciferase activity over media controls. Each time point was performed in triplicate at minimum.

To determine the effects of HDE on downstream pro-inflammatory signal activation in BECs, Cignal Vector Reporters were used. Following reverse transfection of BEAS-2B cells with the luciferase vectors, cells were pre-treated with 0 or 200 nM MaR1 for 1 hour prior to stimulation with HDE. After 1 hour pretreatment, cells were challenged with or without 5% HDE for 1, 6, 12, or 24 hours. In HDE-treated cells, NFκB, AP-1, SP-1, and SRE transcription factor binding activities exhibited significant increases over control cells that received no HDE treatment in a time-dependent manner. No significant changes were seen in the binding activities of NFκB, AP-1, or SP-1 in cells that were given MaR1 pretreatment prior to HDE stimulation (Figure 5A-C). Although, at 24 hours following HDE exposure, there was a significant decrease in SRE activation in MaR1 pretreated cells (Figure 5D). These results suggest that the mechanism by which MaR1 modulates the pro-inflammatory responses of HDE-stimulated BECs is likely not propagated through NFκB, AP-1, and SP-1 transcription factor-related signaling, but may be affecting the activation of SRE-related signaling mechanisms.

## Discussion

Through these investigations, we have found a potential role for the SPM MaR1 in reducing the pro-inflammatory responses of BECs to HDE. These findings include reduced pro-inflammatory cytokine production and PKCα/ϵ activities. In addition, studies performed using a mouse lung slice model indicate the utility of MaR1 in reducing the pro-inflammatory cytokine release caused by HDE in a more complex and biologically relevant model system, where structural and lung resident cells remain in contact, simulating a more realistic biological environment than cell line cultures. Importantly, in the mouse lung slice model, MaR1 was shown to be effective at reducing pro-inflammatory cytokine release in mouse lung slices not only when given as pre-treatment to HDE, but when given 1 hour following HDE exposure as well. These data suggest the potential utility of MaR1 as a preventative as well as a therapeutic treatment in the prevention of airway inflammatory disease.

These findings are of particular importance, as organic dust-related airway inflammation causes multiple deleterious effects. Individuals who experience acute exposures to organic dust develop heightened neutrophilia and pro-inflammatory cytokine production in their airways. Prior studies indicate that BECs play an important role in releasing pro-inflammatory cytokines, which recruit inflammatory leukocytes into the lung following organic dust exposures. Therefore, preventative and treatment measures that reduce the stimulation of these cells by organic dust might prove clinically beneficial to patients. MaR1 may represent a novel class of drugs for preventing and/or treating the airway inflammatory consequences following organic dust exposures.

Recently discovered pro-resolving lipid mediators, such as the resolvins, protectins, lipoxins, and maresins have been found to have potent anti-inflammatory and pro-resolution effects on multiple different cell types that are relevant in the lung environment. For example, resolvin E1 can decrease the levels of IL-6 and IL-1β found in lung tissue following HCl-induced acute lung injury [14]. Resolvin E1 also limited lymphocyte recruitment, IL-13 release, and airway hyper-responsiveness in a murine model of asthma [15]. LPS-induced and cigarette smoke-induced lung inflammation was diminished by treatment with resolvin D1 [16,17]. The role of MaR1 has not yet been characterized within the lung, although studies investigating the effect of MaR1 in limiting inflammation in a murine peritonitis model highlight its potential in attenuating macrophage and neutrophil-dominating inflammatory diseases [18]. Our findings of reduced inflammatory cell-recruiting TNF-α, IL-6, and IL-8/CXCL1 release *in vitro* (BEAS-2B cell line) and/or *ex vivo* (mouse lung slice model) suggests a potential anti-inflammatory role for MaR1 by reducing macrophage and neutrophilic-dominant organic dust-induced lung disease. In our studies, we chose to use concentrations of MaR1 ranging from 1 – 200 nM based upon previous studies with MaR1 and other SPM [18,33], and we found significant effects with as little as 1 nM MaR1. These data suggest that MaR1 can effectively attenuate the HDE-stimulated pro-inflammatory cytokine release in BECs.

**Figure 6:**
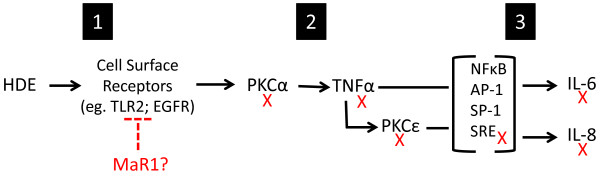
**Summary of known and proposed pro-inflammatory signaling events occurring in BECs following HDE exposure.** The complex composition of HDE leads to activation at multiple surface receptors, including growth factor receptors (i.e. EGFR) and toll-like receptors (i.e.TLR2) **(1)**[35,36]. HDE causes PKCα activity to increase within 1 hr, leading to TNF-α release and activation of PKCϵ by 6 hours **(2)**[8]. IL-6 (dependent on TNF-α) and IL-8 (dependent on PKCϵ) release occurs by 24 hours post-HDE exposure **(3)**[7,8]. Our data implicate activation of NFκB, AP-1, SP-1, and SRE-related transcription factors in the HDE-induced pro-inflammatory response. Our data reported here indicate that MaR1 reduces PKCα and PKCϵ activities, SRE DNA binding activities, and TNF-α, IL-6, and IL-8 release. The direct mechanisms of action of MaR1 on HDE-modulated pathways is undefined but is likely upstream of PKCα activation. Other SPM are known to act through various GPCRs with agonist and antagonist actions, although MaR1 receptor interactions are unknown. A red X indicates a feature of the HDE-induced pro-inflammatory response in BECs that MaR1 reduces.

To better understand the mechanisms underlying the reduced inflammatory cytokine response following organic dust-exposed BECs and mouse lung slices with MaR1 pretreatment, a series of experiments were performed to analyze intracellular signaling events. We have previously reported that HDE-stimulated TNF-α, IL-6, and IL-8 release in BECs is dependent upon the sequential activation of PKC isoforms, where PKCα is responsible for TNF-α release, and TNF-α leads to IL-6 release and PKCϵ activation, while PKCϵ activation subsequently induces IL-8 release [7,8]. A summary of these previously published findings is provided in Figure 6. In this study, pretreatment with MaR1 prior to HDE stimulation effectively abrogated the early (1 hour) activation of PKCα and subsequent TNF-α release, IL-6 release, and activation of PKCϵ at 6 hours. Although PKA activation has been implicated in inhibiting organic dust-induced PKC activation in epithelial cells [32], we observed no change in PKA activity in response to MaR1 pretreatment. Our data suggest that MaR1 interferes with HDE-mediated PKC activation indirectly, as MaR1 appeared to have no enzyme inhibitory effect itself in the presence of the direct PKC activator, PMA. We hypothesize that MaR1 action is upstream of PKCα, potentially at the level of cell surface receptor signaling. As reviewed by Serhan, et al. in 2011, other PRM have been shown to bind specific g-protein coupled receptors to propagate agonist and antagonist effects, and this is a well-known mechanism of action for other poly-unsaturated fatty acid-derived lipid mediators (such as the prostaglandins) [34]. This therefore is likely how MaR1 also acts, although cognate receptors for MaR1 are currently undefined.

Additional potential intracellular signaling/transcriptional pathways were explored to explain the action of MaR1 in reducing pro-inflammatory cytokine release following HDE treatment in BECs. HDE treatment alone temporally activated the binding activities at the NFκB, AP-1, SP-1, and SRE DNA binding elements. Our laboratory and others have previously shown the importance of NFkB and MAPK-related pathway activation in various cells (i.e. phagocytes, epithelial cells) following organic dust exposures [37-40]. MaR1 pretreatment did not significantly affect the total binding activities of NFκB, AP-1, or SP-1. However, MaR1 did attenuate HDE-induced SRE binding activities at 24 hours following HDE exposure. Binding at SRE is known to be activated through both MAPK and Rho kinase signaling mechanisms via serum response factor (SRF) binding at SRE sites [41,42]. This reveals potential pathways that may be targeted by MaR1 to reduce HDE-induced pro-inflammatory consequences to BECs. Interestingly, the activation of SRF-related signaling and SRE binding activity has been shown to be dependent on PKCα and PKCϵ activities [42], corroborating with our findings that these enzymes are required for HDE-related pro-inflammatory cytokine production in BECs and are inhibited by MaR1. The role of SRF-related signaling in airway epithelial cells is not well characterized, nor has this pathway been previously reported to be modulated by SPM. Therefore, future directions will be aimed at investigating the activation of this pathway in BECs, and how the pathway is modulated by MaR1.

It is noteworthy that NFκB activation was not significantly altered by MaR1 treatment in the cells, as reports have shown that omega-3 fatty acids and their derivatives (including resolvin D1, resolvin D5, and resolvin E1), limit NFκB activation, implicating this transcription factor as a key target of various SPM [14,16,43-45]. The lack of alteration in total NFκB activity with MaR1 treatment indicates diversity in the mechanisms by which the various SPM function and highlights the prospects for tailoring specific SPM to different diseases based on their differing activation signatures. Although, it is important to note that NFκB, AP-1, and SP-1 each regulate a substantial number of different gene targets and receive integrative signals to do so [46-48]; it is possible that MaR1 may affect transcriptional activities of specific gene sets regulated by these transcription factors that may cause only subtle changes in total binding activities. Taken together, our data regarding NFκB, AP-1, SP-1, and SRE DNA binding activities suggest MaR1 is acting in part through modulation of SRE-related signaling. Although, we anticipate that MaR1 is also acting through alternate signaling pathways to counterbalance the pro-inflammatory stimulation propagated through these pathways in HDE-stimulated BECs.

## Conclusion

In conclusion, the experiments described in this report indicate a novel role for MaR1 in HDE-induced BEC inflammatory responses whereby MaR1 reduced PKC activation, resulting in diminished epithelial cell TNF-α, IL-6, and IL-8 production following HDE stimulation. DNA binding activity assays revealed MaR1 to have a modulatory effect on HDE-induced SRE-related signaling. Furthermore, studies utilizing the *ex vivo* mouse lung slice model reveal the potential of MaR1 in preventing inflammation when given either prior to *or following* HDE exposure. Together, these data provide support for further investigations aimed at determining the potential utility of MaR1 in limiting organic dust-induced inflammation in an *in vivo* model. A body of work shows that supplementing the diets of patients suffering from acute lung injury or acute respiratory distress syndrome with ω-3 PUFAs significantly lessened lung inflammation and mortality [49]; these studies highlight the potential of SPMs (like MaR1) for treating/ameliorating inflammatory lung conditions. *In vivo* investigations will therefore be highly relevant for determining the potential utility of MaR1 and other SPM in preventing and/or treating organic dust-related exposures in agriculture workers.

## Abbreviations

AP-1: Activator protein-1; BEC: Bronchial epithelial cell; CXCL1: C-X-C motif chemokine ligand 1; HDE: Hog confinement facility-derived organic dust extract; IL-6: Interleukin-6; IL-8: Interleukin-8; MAPK: Mitogen-activated protein kinase; MaR1: Maresin-1; NFκB: Nuclear factor kappa-light-chain-enhancer of activated B cells; PKA: Protein kinase A; PKC: Protein kinase C; PMA: Phorbol 12-myristate 13-acetate; SP-1: Specificity protein-1; SPM: Specialized pro-resolving mediators; SRE: Serum response element; SRF: Serum response factor; TNF-α: Tumor necrosis factor-alpha.

## Competing interests

The authors declare that they have no competing interests.

## Authors’ contributions

TMN contributed to the experimental designs and hypotheses involved in the manuscript, carrying out experimental procedures involved in the manuscript, wrote the manuscript, and contributed to the editing and review of the manuscript. AJH contributed to carrying out experimental procedures involved in the manuscript, and contributed to the editing and review of the manuscript. TAW, JAP, TDL, DRC, and DJR contributed to the experimental designs and hypotheses involved in the manuscript, and contributed to the preparation, editing, and review of the manuscript. All authors read and approved the final manuscript.

## Authors’ information

Contributing Authors’ contact information:

Tara M. Nordgren: 985910 Nebraska Medical Center, Omaha NE 68198

Art J. Heires: 988090 Nebraska Medical Center, Omaha NE 68198

Todd A. Wyatt: 985910 Nebraska Medical Center, Omaha NE 68198

Jill A. Poole: 985300 Nebraska Medical Center, Omaha NE 68198

Tricia D. LeVan: 985910 Nebraska Medical Center, Omaha NE 68198

D. Roselyn Cerutis: 2500 California Plaza, Omaha NE 68178.
